# Secure Data Transfer Based on a Multi-Level Blockchain for Internet of Vehicles

**DOI:** 10.3390/s23052664

**Published:** 2023-02-28

**Authors:** Hua Yi Lin

**Affiliations:** Department of Information Management, China University of Technology, Taipei City 11695, Taiwan; calvan.lin@cute.edu.tw

**Keywords:** roadside unit, intra_clusterBC, inter_clusterBC, ECDSA

## Abstract

Because of the decentralized trait of the blockchain and the Internet of vehicles, both are very suitable for the architecture of the other. This study proposes a multi-level blockchain framework to secure information security on the Internet of vehicles. The main motivation of this study is to propose a new transaction block and ensure the identity of traders and the non-repudiation of transactions through the elliptic curve digital signature algorithm ECDSA. The designed multi-level blockchain architecture distributes the operations within the intra_cluster blockchain and the inter_cluster blockchain to improve the efficiency of the entire block. On the cloud computing platform, we exploit the threshold key management protocol, and the system can recover the system key as long as the threshold partial key is collected. This avoids the occurrence of PKI single-point failure. Thus, the proposed architecture ensures the security of OBU-RSU-BS-VM. The proposed multi-level blockchain framework consists of a block, intra-cluster blockchain and inter-cluster blockchain. The roadside unit RSU is responsible for the communication of vehicles in the vicinity, similar to a cluster head on the Internet of vehicles. This study exploits RSU to manage the block, and the base station is responsible for managing the intra-cluster blockchain named intra_clusterBC, and the cloud server at the back end is responsible for the entire system blockchain named inter_clusterBC. Finally, RSU, base stations and cloud servers cooperatively construct the multi-level blockchain framework and improve the security and the efficiency of the operation of the blockchain. Overall, in order to protect the security of the transaction data of the blockchain, we propose a new transaction block structure and adopt the elliptic curve cryptographic signature ECDSA to ensure that the Merkle tree root value is not changed and also make sure the transaction identity and non-repudiation of transaction data. Finally, this study considers information security in a cloud environment, and therefore we propose a secret-sharing and secure-map-reducing architecture based on the identity confirmation scheme. The proposed scheme with decentralization is very suitable for distributed connected vehicles and can also improve the execution efficiency of the blockchain.

## 1. Introduction

In recent years, the Internet of vehicles has become increasingly mature with the rise of electric vehicles and unmanned self-driving vehicles. In addition, the construction of 5G base stations by various telecom companies has become more popular year by year, which has gradually made the cloudification of the Internet of vehicles (IoV) more feasible, and the Internet of vehicles will be a hot topic, and hundreds of companies must compete in the near future.

The cloud Internet of vehicles can reach vehicles and neighboring equipment to exchange messages with both parties and deliver a large amount of sensing messages to the back-end cloud service platform for big data analysis and computing, generating valuable information. The composition of the Internet of vehicles includes vehicles-to-vehicles communication (vehicles-to-vehicles, V2V), vehicles-to-pedestrian (vehicles-to-pedestrian, V2P), vehicles-to-roadside device (vehicles-to-roadside, V2R), vehicles-to-group telecommunication (vehicles-to-group, V2G), vehicles-to-network (vehicles-to-network, V2N), vehicles-to-infrastructure (vehicles-to-infrastructure, V2I), as well as the vehicles-to-everything (vehicles-to-everything, V2X). The map task and the reduce task have a main cloud server at the back-end called the master, and multiple mapping servers named mapper are responsible for cloud-mapping task services, and the reducer server is responsible for the cloud-reducing task services.

As the vehicle travels, it is able to connect and exchange information with surrounding facilities via V2X and deliver messages to the Internet via roadside device RSUs or base stations. The message is then forwarded through the router to the classifier of the cloud service. Afterward, the classifier assigns the service type of service (ToS) required by the user to the matching cloud service platform. Once the master server of the cloud service platform receives the request, it immediately assigns the mapper server and the reducer server to participate in the operation and performs map/reduce operations according to the requested service.

At this stage, domestic vehicles manufacturers are still in the research stage, and the information of the architecture of the Internet of vehicles has not yet been popularized, but it can be expected that the Internet of vehicles will be one of the key industries for domestic and foreign development in the near future. As is known, the communication protocol of the 802.11P [1] intelligent transportation system proposed by the IEEE organization is an extended version of IEEE 802.11, which is mainly used in vehicles communication security, but there has been little further development after 2009. The newer LTE-V2X technology was proposed in 2015 [2], mainly intended for direct communication between vehicles through LTE, but many technical standards are still under discussion at this stage. However, both 802.11P and LTE-V2X focus on vehicles communications, and there is less discussion about vehicles information security. In the face of the vigorous development of IoVs and cloud computing, it is obvious that it is necessary to further supplement the information security field of the cloud Internet of vehicles in order to deal with the occurrence of information security problems in the near future.

RSUs act as an intermediary bridge and are responsible for transmitting information from the surrounding Internet of vehicles to the back-end base station and cloud service platform. During this data transmission process, we must acknowledge the information security issues between the vehicles OBU to the RSU, the RSU to the base station BS, and the BS to the cloud service platform. Therefore, this study proposes a transport architecture that can cover the information security issues of OBU–RSU–BS–VM communication. After many evaluations, we found that the blockchain used in monetary information security in recent years is quite appropriate for IoVs.

The blockchain has decentralization characteristics; it is difficult to tamper with and forge and contains traceable transaction information. The elliptic curve cryptosystem, ECC, has a fast operation speed, a short key length up to RSA information security strength, and saves computing resources and storage space. Therefore, the blockchain and ECC are very suitable for research relating to the cloud Internet of vehicles. In addition, the information exchange and transaction of the Internet of vehicles will be directly through the exchange of things and no longer reliant on manual transactions, highlighting that the blockchain is very suitable for use in the environment of the Internet of vehicles. Imagine that human beings will no longer need to exit a car, and they can instead communicate with a fuel dispenser through the OBU, or vehicle cleaning equipment connections and a variety of short-range Internet of things device communications and transactions. The blockchain will play an extremely important role in this process; therefore, we want to develop the information security of IoVs based on the blockchain.

The rest of the present document is structured as follows. An overview of the associated research is presented in Section 2. Section 3 presents the proposed secure data transfer based on a multi-level blockchain framework for the Internet of vehicles. Additionally, this section introduces the transaction block and the ECDSA digital signature, and the information security transmission method. Additionally, the secure mapped/reduced data transmission agreement is presented. The analysis of performance and security is provided in Section 4. Finally, conclusions and subsequent work are explained in Section 5.

## 2. Related Work

**Figure 1:**
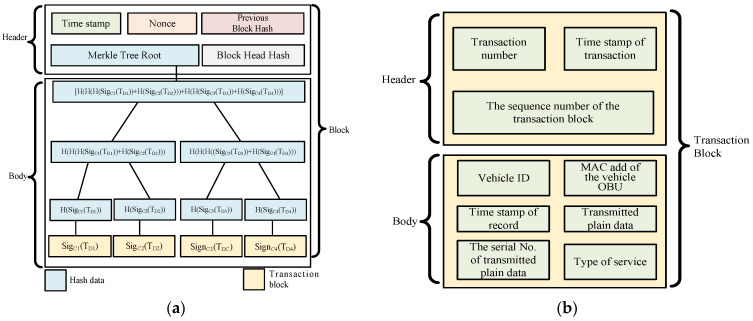
The detailed structure of the block. (**a**) The transitional block. (**b**) The proposed transaction block.

The blockchain is a peer-to-peer distributed database. In contrast to traditional databases, the data are reposited in a primary location, while the blockchain spreads these data across many secondary locations, which are referred to as nodes. In addition, the blockchain has several characteristics: 1. Decentralization 2. Anonymous 3. Prevent tampering 4. Data consistency 5. Information transparency [3,4]. A blockchain is made up of many combined blocks, and then those blocks are tensed together into a blockchain. Additionally, every block consists of two kinds of information, namely the block header and the block body. Figure 1a demonstrates the structure of the complete block; the types of data below are block headers [5,6].

(1) Pervious block hash: The prev_hash value is the hash calculated from the block header of the preceding block.
(2) Timestamp: Generate the timestamp of this block.
(3) Nonce: This represents how many workload algorithms there are and how difficult these algorithms are.
(4) Merkle tree root hash: This represents the value of the hash operation of the current block body and the hash value of the Merkle root node that is computed according to the algorithm of the Merkle tree.

We consider the Merkle tree to be an arborescent structure. Every non-leaf node has a hash value. This study exploits this tree architecture to obtain the hash value of the data, and the timestamp indicates when the block was generated and makes sure that every block is sequentially linked. Moreover, the root of the Merkle tree is the aforementioned root node. The child nodes below it are all the transaction events that occur. The block body is the same as all the information about the transaction. Additionally, the transaction is the message of the generated block body, including the creation time, data and the size of the record, received transaction number, the hash value of the transaction of the Merkle tree node, the digital signature of the transaction, the transaction identification address, and the transaction record’s index number, which is conducive to querying the address of the transaction. Each transaction is linked to a hash value to form a node in the Merkle tree to make sure that the transaction cannot be copied or tampered with. In addition, the blockchain has the following characteristics.

(1) Genesis block:

It is located in the first block, and the value of the field prev_hash in this block is null. Once a blockchain is generated, it starts by creating a genesis block. Other blocks make use of the previous block called the prev_hash field within the header to store the hash value of the preceding block header and obtain an entire blockchain.

(2) The blockchain is not able to be altered:

As a result of changing the transaction records, the value of the Merkle tree root in the block header will be altered, causing the prev_hash field values of each block header, which are concatenated by the entire blockchain, to change synchronously because the integrity of the blockchain is broken. As a result, the prev_hash field values of the previous block that led to the next block must be adjusted in the same way; therefore, if someone wants to modify the transaction history of the block, they must modify all of the following blocks, which is almost impossible to perform.

Looking around in recent years, the majority of the proposed methods have focused on the security of the IoVs but have lacked the integration of back-end cloud service platforms. Many of the proposed methods concentrate on how to deal with secure transmission in terms of the IoVs. After surveying a variety of research papers and discussions, this study summarizes the information security mechanism of the IoVs proposed at this stage in several directions: (1) The blockchain in business transactions and management model; (2) interchain and intrachain architectures; (3) the integration of the blockchain in various network layers; (4) a privacy-preserving authentication blockchain for vehicle ad hoc networks; (5) the consortium blockchain; (6) the batch authentication protocol of the blockchain-based IoT; (7) an anonymous authentication mechanism in the blockchain; (8) a lightweight authentication based on the blockchain. The detailed descriptions are as follows:

(1) In 2019, Jiang et al. [7] divided the application of the blockchain in business transactions and management models into five categories, namely vehicle management blockchain, vehicle manufacturing blockchain, user privacy blockchain, vehicle insurance purchase blockchain and the common data blockchain. Shrestha et al. [8] proposed that a regional blockchain can achieve an attack success rate of 51% by controlling several control parameters, such as the number of vehicles, malicious vehicles, messaging and time and puzzle calculations under the premise of ensuring stability.
(2) In 2019, Ma et al. [9] proposed a privacy-secure and decentralized vehicle network architecture. In the architecture, RSUs play the role of the main blockchain storage node. In addition, the cloud computing node is in charge of storing and backing up the data of the blockchain. Moreover, this architecture consists of two distinct sub-blockchains, called inter-blockchain and intra-blockchain. Interchain is in charge of communicating information between RSUs, vehicles and infrastructure. The intrachain supports sensors that allow drivers to communicate with passengers in the car.
(3) In 2020, Dai et al. and Lu et al. [10,11], based on a ledger structure, set up a private blockchain to store transactions on a secure communication network with crowdsourcing tasks. Overcoming the traditional crowdsourcing single-point error problem, Liu et al. [12] incorporated blockchain mechanisms at the data layer, network layer, application layer, AI layer and business layer. Among them, the network layer contains the peer-to-peer network sublayer and the collaborative network module of the blockchain. Moreover, the AI layer consists of the consensus sublayer of the blockchain, the analysis services and vehicle-oriented computing, including the block consensus protocol executed at this layer.
(4) Lu et al. [13] offered a blockchain-based VANETs (vehicular ad hoc networks) privacy protection authentication protocol in 2019 called the BPPA protocol. The authors developed a privacy-preserving authentication blockchain for VANETs. The proposed BPPA scheme uses a blockchain to remain immutable and store all credentials and transactions to achieve transparency and verifiability of TAs. In addition, this research provides a mechanism capable of distributed authentication but does not need a revocation list. To achieve indirect privacy, the study authorizes vehicles to deploy credentials that are encrypted and retained in the blockchain. If there is an inconsistency, it can be disclosed through a link.
(5) In 2022, Cui et al. [14] designed an effective data-sharing method between vehicles named the consortium blockchain. In traditional vehicle systems, data sharing takes place between the vehicle and roadside equipment. However, the authors used a distributed technology consortium to enable the sharing of traceable information between anonymous vehicles. Furthermore, the combination of 5G and blockchain makes it possible to share data with no RSUs.
(6) In 2021, Bagga and other scholars [15] proposed a batch authentication protocol for blockchain-based IoT. There are two types of authentication: (1) vehicle-to-vehicle authentication. In a cluster, this mode allows the authentication of a vehicle with adjacent vehicles. (2) Batch authentication enables the same group of vehicles to authenticate via their RSUs. Ultimately, cluster vehicles and RSUs can collaborate to establish a group key.
(7) In 2021, Maria et al. [16] proposed an anonymous authentication mechanism, which can be applied to the security of the vehicular ad hoc networks during the switching process between the vehicles and the roadside device RSU that consumes fewer computing resources at reduced costs.
(8) In 2022. Zheng et al. [17] offered a lightweight blockchain-based authentication and an IoT key agreement to improve the effectiveness of the authentication using a multi-TA model. The authors used the blockchain to save the vehicle’s authentication information and cross-region authentication to protect the user’s private information. At the same time, the proposed method adopts lightweight computing to shorten the certification time of the vehicles and complete the whole certification procedure.

In summary, most research has focused on connected car networks [18,19], wherein the vehicles’ collected data are finally delivered to the back-end cloud service platform for big data analysis and processing so as to acquire valuable information. Consequently, the aforementioned research lacks a discussion of the security transmission mechanisms of IoVs combined with back-end cloud computing information. In view of this, we designed an information security mechanism based on blockchain combined with front-end vehicle terminal equipment and a back-end cloud service platform.

## 3. A Secure Data Transfer Based on a Multi-Level Blockchain Framework for Internet of Vehicles

### 3.1. The Transaction Block and the ECDSA Digital Signature for IoVs

Due to the huge number of vehicles in the IoVs and the rapidly changing topology, we proposed a customized cloud IoV transaction block and a digital signature for the onboard transaction block through the ECDSA scheme, which can ensure the integrity and non-repudiation of transaction data, thus protecting the transmission security of vehicle transaction information. The proposed transaction block’s internal structure contains the following information, as shown in Figure 1b.

Transaction block header

(1) Transaction number: the serial number of the transaction.
(2) Timestamp of the transaction: when the transaction block was produced.
(3) The sequence number of the transaction block: the sequence of the transaction block that is generated.

Transaction block body

(1) Vehicle ID: Identification of the vehicle.
(2) MAC add of the vehicle OBU: the vehicle hardware manufacturing number.
(3) Timestamp of record: the time in which the transaction record is generated.
(4) Transmitted plain data: textual information to be transmitted by vehicles.
(5) The serial number of the transmitted plain data: the amount of data to be transmitted by the vehicles.
(6) Type of service: The category of cloud service required for the vehicle.

In addition, this study considers the integrity and non-repudiation of the transaction data; we exploit the ECDSA to achieve the aforementioned functions.

ECDSA signature procedures

**Figure 2:**
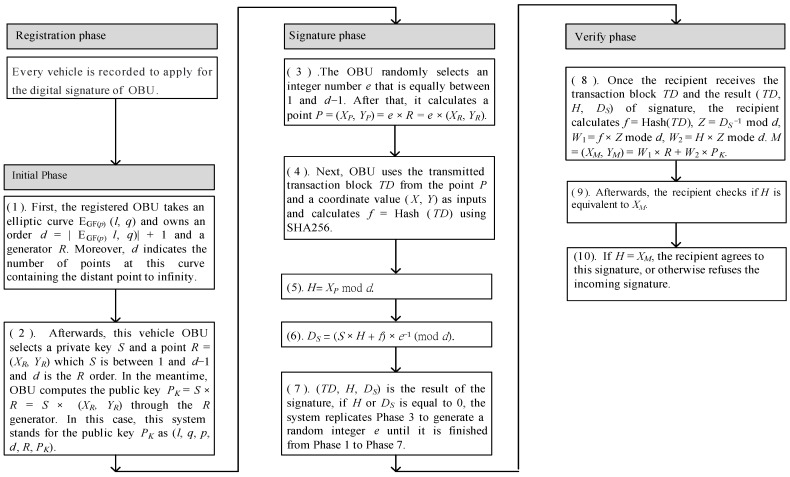
The procedure of the ECDSA digital signature on the transaction block.

When the vehicles need to transmit data, the transaction block must be digitally signed by the ECDSA to assure the integrity and non-repudiation of the transaction data [20,21]. Here, we introduce the elliptic curve cryptosystem into the OBU and RSU, and the detailed signature process is described below, as shown in Figure 2.

Initially phase:

Phase 1. First, the registered OBU takes an elliptic curve E_GF(*p*)_ (*l*, *q*) and owns an order *d* = |E_GF(*p*)_ *l*, *q*)| + 1 and a generator *R*. Moreover, *d* indicates the number of points on this curve containing the distant point to infinity.
Phase 2. Afterward, this vehicle OBU selects a private key S and a point *R* = (*X_R_*, *Y_R_*); *S* is between 1 and *d* − 1, and *d* is the *R* order. In the meantime, the OBU computes the public key *P_K_* = *S* × *R* = *S* × (*X_R_*, *Y_R_*) through the *R* generator. In this case, this system stands for the public key *P_K_*, as (*l*, *q*, *p*, *d*, *R*, *P_K_*).

Signature phase:

Phase 3. The OBU randomly selects an integer number e that is equally between 1 and *d* − 1. After that, it calculates a *P* point = (*X_P_*, *Y_P_*) = *e* × *R* = *e* × (*X_R_*, *Y_R_*).
Phase 4. Next, the OBU uses the delivered transaction block *TD* coming from the point *P* and a coordinate value (*X*, *Y*) as inputs and calculates *f* = Hash (*TD*) using SHA256.
Phase 5. *H* = *X_P_* mod *d*.
Phase 6. *D_S_* = (*S* × *H* + *f*) × *e*^−1^ (mod *d*).
Phase 7. (*TD*, *H*, *D_S_*) is the result of the signature; if *H* or *D_S_* is equal to 0, the system replicates Phase 3 to generate an arbitrary integer e until it is accomplished from Phase 1 to Phase 7.

Verify phase

Phase 8. Once the recipient receives the transaction block *TD* and the result (*TD*, *H*, *D_S_*) of the signature, the recipient calculates *f* = Hash (*TD*), *Z* = *D_S_*^−1^ mod *d*, *W*_1_ = *f* × *Z* mode *d*, *W*_2_ = *H* × *Z* mode *d*. *M* = (*X_M_*, *Y_M_*) = *W*_1_ × *R* + *W*_2_ × *P_K_*.
Phase 9. Afterward, the recipient checks if *H* is equivalent to *X_M_*.
Phase 10. If *H* = *X_M_*, the recipient agrees to this signature or otherwise refuses the incoming signature.

Secure phase

If the vehicles want to deliver data to the cloud service platform, first, the vehicles need to execute the ECDSA digital signature procedure on the transaction block to obtain the digital signature result.

The architecture based on a multi-level security management

**Figure 3:**
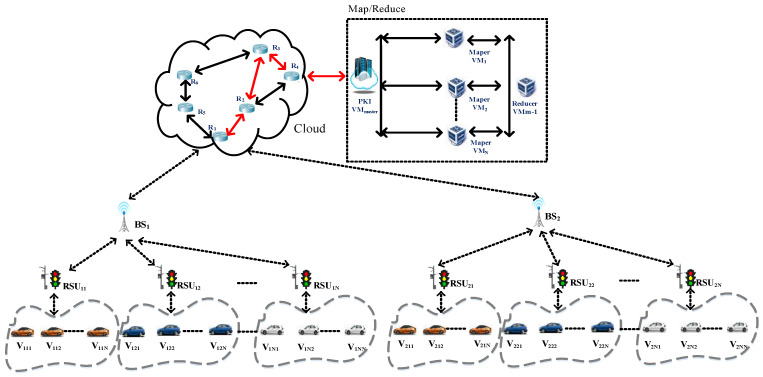
The multi-level security architecture.

The IoVs integrates the topology logic of onboard, roadside devices and cloud-side servers. Since the Internet of vehicles changes rapidly, here we consider the information security efficiency of the IoVs. Different from the traditional blockchain framework, the designed multi-level blockchain architecture is divided into three layers as an edge computing architecture. Each layer is in charge of its own tasks and performs the operation of the blockchain. Thus, the proposed architecture can handle the latency issues of a traditional block. In this system, each level of security is similar to a layered delegation of authority, and each level performs its own responsibilities. The designed multi-layered security architecture of the Internet of vehicles blockchain mechanism is shown in Figure 3. This architecture is an intra-blockchain based on the M-Tree dynamic management of each base station to deal with the security issues of all levels of the IoVs [21]. Since the vehicle is located at the bottom of the multi-level security architecture of the Internet of vehicles blockchain system, we lay out the vehicle to the leaf node. Additionally, RSUs manage the transaction block transmitted from the vehicle’s OBU. In this study, RSUs are placed on the third level of the security level, the base station (local credential authorization) is placed at the second layer, and the cloud server VMmaster (global credential authorization) corresponds to the upper layer of the security level, which is the top layer and is responsible for cooperating with different base stations to construct an inter-blockchain.

This study takes into account the fact that the OBU devices equipped in vehicles generally do not have strong computing capabilities. Before deployment, we verify and register the BSs and RSUs; therefore, if nonregistered BSs and RSUs want to join the operation, this system will turn them down. In this study, RSUs are responsible for the communication transmission of the transaction blocks with surrounding vehicles, and the base stations BSs with more powerful computing functions are responsible for cooperating with different RSUs to construct an intra-blockchain, while the back-end cloud PKI is in charge of combining the intra-blockchains into a complete inter-blockchain for this IoVs system.

The proposed architecture can efficiently enhance the performance of the overall blockchain and reduce the synchronization time of the blockchain. Additionally, we adopt the ECDH key exchange protocol to compute the common conference key between the routers and encrypt/decrypt the transmitted data to ensure the security of the information transmission among the base station, routers and the PKI VM_*master*_.

To this end, a multi-level blockchain management protocol is proposed, and the elliptic curve signature cryptosystem is embedded into the vehicles’ OBU and roadside devices’ RSUs [21]. When the transaction message is transmitted by the vehicles, we adopt the transaction block and sign the transaction block through ECDSA to ensure the non-repudiation of the transaction and ensure the integrity of the transaction data.

**Figure 4:**
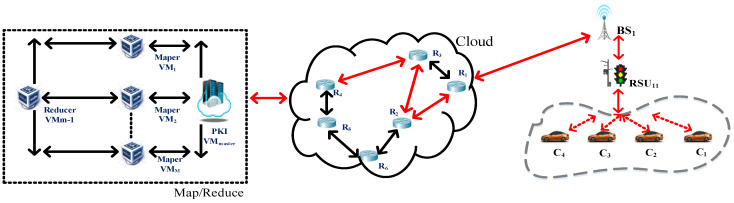
The routing path of the transaction block.

Herein, this study assumes that when the moving vehicles pass through the path *C*_1_ ←→ *C*_2_ ←→ *C*_3_ ←→ *C*_4_ and transmit the transaction, the blocks are *T_D_*_1_~*T_D_*_4_, as shown in the red line of Figure 4. This study employs blockchain algorithms to protect the delivered transaction block. The detailed procedure is described below, and the usage notation in this system is represented in Table 1.

**Figure 5:**
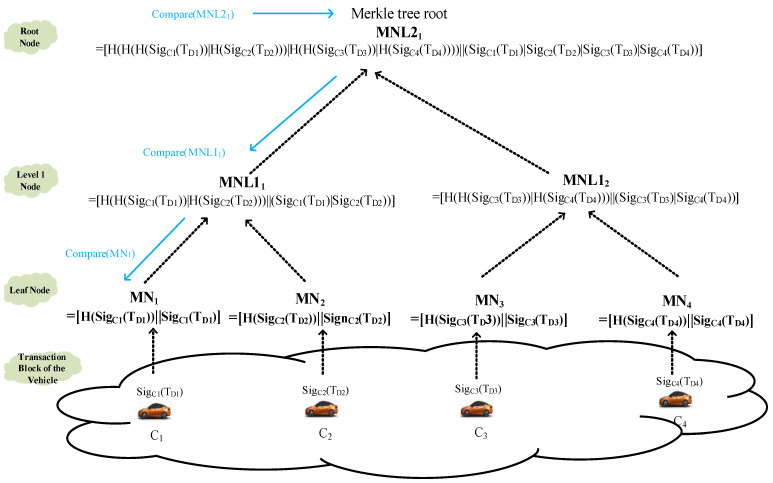
The calculation and verification of the Merklet root hash value.

Phase 1: First, the RSU is responsible for calculating the internal cluster blockchain named intra_clusterBC.
  Step 1. In this study, ECDSA digital signatures are used to digitally sign the transaction block. First, each vehicle *C*_1_~*C*_4_ performs an ECDSA signature on all of the transmitted transaction blocks *T_D_*_1_~*T_D_*_4_. Subsequently, we adopt SHA256 to calculate the hash value of each vehicle’s transaction block signature. Subsequently, H(Sig*_C_*_i_(*T_Di_*)) is obtained, and the digital signature result is mapped to the Merkle tree leaf node MN*_i_* = [H(Sig*_Ci_*(*T_Di_*))||Sig*_Ci_*(*T_Di_*)], *i* = 1~4, as shown in Figure 5. For example, MN_1_ = [H(Sig*_C_*_1_(*T_D_*_1_))||Sig*_C_*_1_(*T_D_*_1_)], MN_2_ = [H(Sig*_C_*_2_(*T_D_*_2_))||Sig*_C_*_2_(*T_D_*_2_)], MN_3_ = [H(Sig*_C_*_3_(*T_D_*_3_))||Sig*_C_*_3_(*T_D_*_3_)], MN_4_ = [H(Sig*_C_*_4_(*T_D_*_4_))||Sig*_C_*_4_(*T_D_*_4_)].
  Step 2. Afterward, we combine two nearby Malekle nodes together and execute the hash operation to obtain the parent node at level 1 MNL1_[(*i*+1)/2]_ = [H(H(Sig*_C__i_*(*T_Di_*))|H(Sig*_C__i_*_+1_(T_Di+1_)))||Sig*_C__i_*(*T_Di_*)|Sig*_C__i_*_+1_(*T_Di_*_+1_)], *i* = 1, 3, 5, 7,….
  Step 3. The above similar steps are repeated to combine two nearby parent nodes and execute the hash operation to obtain the ancestor node at level 2 MNL2_[(_*_j_*_+1)/2]_ = [H(H(H(Sig*_C_*_i_(*T_Di_*))|H(Sig*_C__i_*_+1_(*T_D__i_*_+1_)))|H(H(Sig*_C__i_*_+2_(*T_D__i_*_+2_))|H(Sig*_C__i_*_+3_(*T_D__i_*_+3_))))]|| H(Sig*_C_*_i_(*T_Di_*)|Sig*_C__i_*_+1_(*T_D__i_*_+1_)|Sig*_C__i_*_+2_(*T_D__i_*_+2_)|Sig*_C__i_*_+3_(*T_D__i_*_+3_) ], *i* = 1, 5, 9,…, j = 1, 3, 5, 7,….
  Step 4. Repeat step 2 until the system obtains the Markle tree root node, as shown by the black dotted line in Figure 5.

Phase 2: The base station BS connects the blockchains named intra_clusterBC, which are formed by the RSU, and there is a corresponding RSU responsible for the connected vehicles. Since each RSU is in charge of the management of the block within the cluster, this study assigns RSU*_ix_*, respectively, where *i* represents the level, and *x* represents the number of the RSU and also the intra_clusterBC*_x_*.

**Figure 6:**
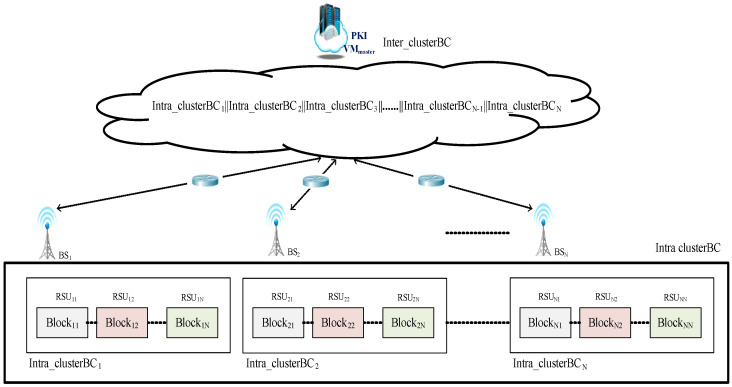
The multi-level blockchain framework for Internet of vehicles.

**Figure 7:**
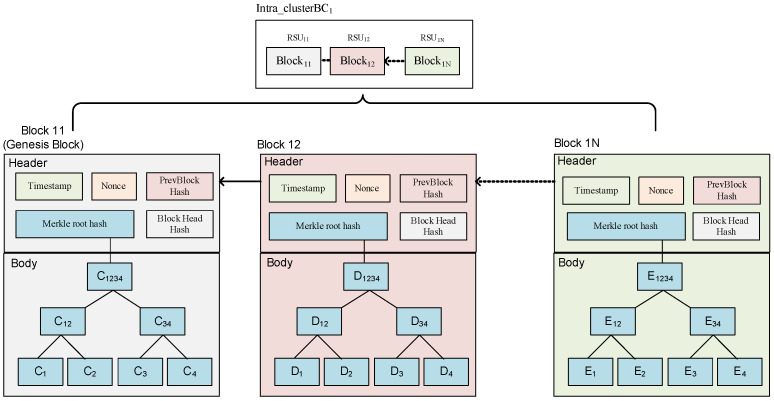
The detailed structure of the intra_clusterBC.

Additionally, multiple vehicles in each RSU*_ix_* transmit their signed transaction block, the block name is Block*_ix_*, and the block is managed by RSU*_ix_*_._ Afterward, BS*_i_*, where *i* represents the number of the base station, connects the intra_clusterBC*_i_* formed by the RSU*_ix_*, as shown in Figure 6 and Figure 7.

Phase 3: The cloud service platform PKI VM*_master_* combines each intra_clusterBC*_i_* coming from BS*_i_* to form inter_clusterBC, as shown in Figure 6.

Finally, the cloud service platform PKI VM*_master_* must integrate and manage the intracluster blockchain sent back by all the base stations. Since the cloud service platform VM*_master_* is located in the top layer of the whole system, the intra_clusterBC*_i_* is managed by BS*_i_* must be concatenated to form the intercluster blockchain named inter_clusterBC of the whole system, as shown in Figure 6.

### 3.2. Information Security Transmission Method between IoVs and Routers

When the intra_clusterBC*_x_* is complete, the base station BS*_x_* is responsible for transmitting the intra_clusterBC*_x_* to the cloud service platform VM*_master_*, and the transmission process must pass through the router to protect the intra_clusterBC*_x_* information. This study adopts the elliptic curve cryptographic exchange protocol, ECDH, to protect the transmitted data. Here, we assume that the base station BS*_x_* and router are secure and certified before deployment. The delivered data are routed through the *BS*_1_→*R*_1_→*R*_2_→*R*_3_→*R*_4_→PKI VM*_master_* path, as shown by the red line in Figure 4.

Initially, the vehicle digitally signs the transaction block using ECDSA and then performs a hash operation through the RSU to obtain the hash value of the Merkle tree root and the complete block. When each RSU performs similar operations and individually transmits its blocks to the BS, the BS can concatenate the received blocks into an intra_clusterBC. The router then transfers the intra_clusterBC to the destination, VM*_master_*. In this study, ECDH key agreement is used on the routing side, and the two parties use the ECDH conference key to cipher and decipher the transmitted data [21]. The ECDH mechanism is similar to the traditional Diffie–Hellman key exchange protocol, where both sides set up a conference key over an unsecured channel [22]. Since asymmetric cryptosystems have a key length of at least 1024 bits, they offer an elevated grade of security. However, ECDH utilizes the key protocol of Diffie–Hellman to implement elliptic curve cryptosystems that require merely a 160-bit key strength and use less computing power to achieve similar security intensity [23,24]. Therefore, it is highly adapted to the Internet of vehicles that are short of computer capabilities. Similarly, in this study, the information security transmission between the router and the cloud service platform can also be protected by ECDH.

Cloud information security transmission mechanism

When *BS*_1_ receives the intra_clusterBC_1_, *BS*_1_ and *R*_1_ cooperate to figure out the common conference key SK*_BS_*_1_*R*1_ using the ECDH key exchange protocol, and then *BS*_1_ and *R*_1_ adopt SK*_BS_*_1_*R*1_ to encrypt and protect the timestamp, sequence number, routing path, and intra_clusterBC_1_. Then, the encrypted result is delivered to the *R*_1_ router.

#*BS*_1_→*R*_1_

EN_SK_*_BS_*_1_*R*1_[(*BS*_1_)|*SN*|*TS*|intra_clusterBC_1_]

When the router *R*_1_ receives the message, it decrypts the accepted encrypted message through the common conference key of SK*_BS_*_1_*R*1_ and adds its identity ID to the passing path. Then, depending on the routing table, *R*_1_ and the following router *R*_2_ use the ECDH protocol to jointly calculate the conference key of SK*_R_*_1_*R*2_, and then *R*_1_ encrypts the entire message and transmits the encryption result to the *R*_2_ router.

#*R*_1_→*R*_2_

EN*_SK__R_*_1_*R*2_[(*R*_1_, *BS*_1_)|*SN*|*TS*|intra_clusterBC_1_]

In the same way, when the router R_2_ receives the message, it decrypts the accepted encrypted message through the common conference key of SK_R1_R2_ and adds its own ID to the passing path. Then, depending on the routing table, R_2_ and the following router R_3_ use the ECDH protocol to jointly calculate the conference key of SK_R2_R3_, and then R_1_ encrypts the entire message and transmits the encryption result to the R_2_ router. Therefore, it repeats until the message is delivered to the PKI VM_*master*_.

#*R*_2_→*R*_3_

EN*_SK__R_*_2_*R*3_[(*R*_2_, *R*_1_, *BS*_1_)|*SN*|*TS*|intra_clusterBC_1_]

#*R*_3_→*R*_4_

EN*_SK__R_*_3_*R*4_[(*R*_3_, *R*_2_, *R*_1_, *BS*_1_)|*SN*|*TS*|intra_clusterBC_1_]

#*R*_4_→PKI VM*_m_*

EN*_SK__R_*_4_VM*master*_[(*R*_4_, *R*_3_, *R*_2_, *R*_1_, *BS*_1_)|*SN*|*TS*|Intra_clusterBC_1_]

When VM*_master_* receives the encrypted message, it immediately uses the SK*_R_*_4_VM*master*_ to decrypt the encrypted message to obtain intra_clusterBC_1_, and so on to obtain the cluster blockchain from BS_2_~BS*_N_*, where there are intra_clusterBC_1_~intra_clusterBC*_N_*, and then they are concatenated together to become a complete inter_clusterBC.

Under special circumstances, such as vehicle emergencies wherein the vehicle needs to transmit data immediately, only RSU_1_ is responsible for clustering the internal vehicles to transmit the transaction information in a single block. In order to facilitate the explanation of the block transmitted by this single RSU_1_ for explanation, we assume that RSU_1_ contains vehicles *C*_1_~*C*_4_. To guarantee security during data transmission, first, *BS*_1_ and *R*_1_ calculate the conference key of SK*_BS_*_1_*R*1_ between each other via the ECDH key exchange agreement, and subsequently, *BS*_1_ encrypts and protects the routing path, sequence number, timestamp stamp and the Merkle tree root HMAC value in the block, concatenating the original data, and then transmitting the encrypted result to the *R*_1_ router.

#*BS*_1_→*R*_1_

EN_SK_*_BS_*_1_*R*1_[(*BS*_1_)|*SN*|*TS*|[H(H(H(Sig*_C_*_1_(*T_D_*_1_))|H(Sig*_C_*_2_(*T_D_*_2_)))|H(H(Sig*_C_*_3_(*T_D_*_3_))|H(Sig*_C_*_4_(*T_D_*_4_))))||(Sig*_C_*_1_(*T_D_*_1_)|Sig*_C_*_2_(*T_D_*_2_)|Sig*_C_*_3_(*TD*_3_)|Sig*_C_*_4_(*T_D_*_4_))]]

When *R*_1_ receives the message, the two parties can decrypt the accepted encrypted message because they have a common conference key of SK*_BS_*_1_*R*1_. Then, its own ID is appended to the passing path, and referring to the routing table, *R*_1_ and the following router *R*_2_, the two parties cooperate via the ECDH key exchange agreement to calculate the conference key of SK*_R_*_1_*R*2_, and then *R*_1_ encrypts the entire message[(*R*_1_, *BS*_1_)|*SN*|*TS*|[H(H(H(Sig*_C_*_1_(*T_D_*_1_))|H(Sig*_C_*_2_(*T_D_*_2_)))|H(H(Sig*_C_*_3_(*T_D_*_3_))|H(Sig*_C_*_4_(*T_D_*_4_))))||(Sig*_C_*_1_(*T_D_*_1_)|Sig*_C_*_2_(*T_D_*_2_)|Sign*_C_*_3_(*T_D_*_3_)|Sign*_C_*_4_(*T_D_*_4_))]], transmitting the encryption result to the *R*_2_ router.

#*R*_1_→*R*_2_

EN_SK_*_R_*_1_*R*2_[(*R*_1_,*BS*_1_)|*SN*|*TS*|[H(H(H(Sig*_C_*_1_(*T_D_*_1_))|H(Sig*_C_*_2_(*T_D_*_2_)))|H(H(Sig*_C_*_3_(*T_D_*_3_))|H(Sig*_C_*_4_(*T_D_*_4_))))||(Sig*_C_*_1_(*T_D_*_1_)|Sig*_C_*_2_(*T_D_*_2_)|Sig*_C_*_3_(*T_D_*_3_)|Sig*_C_*_4_(*T_D_*_4_))]]

When R_2_ obtains the data transmitted by R_1_, both parties decrypt the encrypted data through SK_R1_R2_, the common conference key, and then R_2_ adds its ID to the passing path. Then, depending on the routing table, R_2_ and the following router, R_3_, work together to calculate the conference key of SK_R2_R3_ using the ECDH key exchange protocol, and then R_2_ use SK_R2_R3_ to encrypt the entire message [(R_2_, R_1_, BS_1_)|SN|TS |[H(H(H(Sig_C1_(T_D1_))|H(Sig_C2_(T_D2_)))|H(H(Sig_C3_(T_D3_))|H(Sig_C4_(T_D4_))))||(Sig_C1_(T_D1_)|Sig_C2_(T_D2_)|Sig_C3_(T_D3_)|Sig_C4_(T_D4_))]], transmitting the encrypted result to the R_3_ router.

#*R*_2_→*R*_3_

EN*_SKR_*_2_*R*3_[(*R*_2_, *R*_1_, *BS*_1_)|*SN*|*TS* |[H(H(H(Sig*_C_*_1_(*T_D_*_1_))|H(Sig*_C_*_2_(*T_D_*_2_)))|H(H(Sig*_C_*_3_(*T_D_*_3_))|H(Sig*_C_*_4_(*T_D_*_4_))))||(Sig*_C_*_1_(*T_D_*_1_)|Sig*_C_*_2_(*T_D_*_2_)|Sig*_C_*_3_(*T_D_*_3_)|Sig*_C_*_4_(*T_D_*_4_))]]

Similarly, when *R*_3_ obtains the data transmitted by *R*_2_, both parties calculate the common conference key of SK*_R_*_2_*R*3_, decrypt the received encrypted data, and append its own ID to the passing path. Afterward, based on the routing table, *R*_3_ and the following router, R_4_, calculate the common conference key of SK*_R_*_3_*R*4_ via the ECDH key exchange agreement, and then *R*_3_ uses the SK*_R_*_3_*R*4_ to cipher the entire message [(*R*_3_, *R*_2_, *R*_1_,*BS*_1_)|*SN*|*TS*|[H(H(H(Sig*_C_*_1_(*T_D_*_1_))|H(Sig*_C_*_2_(*T_D_*_2_)))|H(H(Sig*_C_*_3_(*T_D_*_3_))|H(Sig*_C_*_4_(*T_D_*_4_))))||(Sig*_C_*_1_(*T_D_*_1_)|Sig*_C_*_2_(*T_D_*_2_)|Sig*_C_*_3_(*T_D_*_3_)|Sig*_C_*_4_(*T_D_*_4_))]], subsequently delivering the enciphered result to the router *R*_4._

#*R*_3_→*R*_4_

EN*_SK__R_*_3_*R*4_[(*R*_3_, *R*_2_, *R*_1_, *BS*_1_)| *SN*|*TS* |[H(H(H(Sig*_C_*_1_(*T_D_*_1_))|H(Sig*_C_*_2_(*T_D_*_2_)))|H(H(Sig*_C_*_3_(*T_D_*_3_))|H(Sig*_C_*_4_(*T_D_*_4_))))||(Sig*_C_*_1_(*T_D_*_1_)|Sig*_C_*_2_(*T_D_*_2_)|Sig*_C_*_3_(*T_D_*_3_)|Sig*_C_*_4_(*T_D_*_4_))]]

#*R*_4_→*R*_5_

EN*_SKR_*_4_*R*5_[(*R*_4_, *R*_3_, *R*_2_, *R*_1_, *BS*_1_)|*SN*|*TS* |[H(H(H(Sig*_C_*_1_(*T_D_*_1_))|H(Sig*_C_*_2_(*T_D_*_2_)))|H(H(Sig*_C_*_3_(*T_D_*_3_))|H(Sig*_C_*_4_(*T_D_*_4_))))||(Sig*_C_*_1_(*T_D_*_1_)|Sig*_C_*_2_(*T_D_*_2_)|Sig*_C_*_3_(*T_D_*_3_)|Sig*_C_*_4_(*T_D_*_4_))]]

The above steps are repeated, and *R*_4_ and *R*_5_ obtain the transmitted encrypted message; then, SK*_R_*_3_*R*4_ and SK*_R_*_4_*R*5_ decrypt the encrypted message through the common conference key and append their own ID to the routing path. Subsequently, *R*_5_ finds the destination of VM*_master_* according to the routing path table, and then the two parties use ECDH to jointly calculate the conference key SK*_R_*_5_VM*master*_, and then *R*_5_ uses the SK*_R_*_5_VM*master*_ to encrypt the entire message [(*R*_5_, *R*_4_, *R*_3_, *R*_2_, *R*_1_, *BS*_1_)|*SN*|*TS*|[H(H(H(Sig*_C_*_1_(*T_D_*_1_))|H(Sig*_C_*_2_(*T_D_*_2_)))|H(H(Sig*_C_*_3_(*T_D_*_3_))|H(Sig*_C_*_4_(*T_D_*_4_))))||(Sig*_C_*_1_(*T_D_*_1_)|Sig*_C_*_2_(*T_D_*_2_)|Sig*_C_*_3_(*T_D_*_3_)|Sig*_C_*_4_(*T_D_*_4_))]] and send the encrypted result to VM*_master_*.

#*R*_5_→PKI VM*_master_*

EN_SK*R*5_VM*master*_[(*R*_5_, *R*_4_, *R*_3_, *R*_2_, *R*_1_, *BS*_1_)|*SN*|*TS* |[H(H(H(Sig*_C_*_1_(*T_D_*_1_))|H(Sig*_C_*_2_(*T_D_*_2_)))|H(H(Sig*_C_*_3_(*T_D_*_3_))|H(Sig*_C_*_4_(*T_D_*_4_))))||(Sig*_C_*_1_(*T_D_*_1_)|Sig*_C_*_2_(*T_D_*_2_)|Sig*_C_*_3_(*T_D_*_3_)|Sig*_C_*_4_(*T_D_*_4_))]]

Upon receiving the data, PKI VM*_master_* deciphers the enciphered data through the SK*_R_*_5_Vm*master*_, confirms the service type required by the TS (type of service) field inside each onboard transaction block, and then transmits the data to the correlative cloud service server to perform the emergency service required by the vehicle. Since the PKI VM*_master_* is the master VM of the cloud service platform [21], the VM*_master_* subsequently continues to perform mapping/reduction tasks.

### 3.3. The Secure Data Transmission Agreement for Map/Reduce

When the transaction block of the vehicle is transmitted to the cloud platform to perform mapping/reduction operations, it may be attacked by malicious VMs. Therefore, this study considers the certainty and security of the identity of VMs that have joined operations to avoid identity spoofing. When the reduction operation reads the data from the mapper, it must also confirm the integrity of the data and confirm the identity of the mapper so as to avoid malicious modifications of the data and read the data transmitted by the malicious VMs. In summary, this study proposes that the group signature and threshold key protection mechanism perform secure mapping and reduction operations, mainly using the secret sharing method proposed by Shamir and Blakley [25]. This mechanism contains two essential parameters. There is the threshold value, *n*, and the number of shared keys, *m*, which are generally expressed as (*m*, *n*), and this method has a secret fault tolerance mechanism.

First, the system distributes the selected master key into *m* different sharing keys, and each computer participating in the cloud computing obtains a shared key, and when the number of shared keys obtained is greater than or equal to the *n* value, the master key can be recovered. However, when the number of shared keys obtained is less than *n*, the master key cannot be recovered.

In the proposed model, the PKI VM*_master_* computer first calculates the system key, *S*, and its corresponding public key, *P*, and then divides the system key into *m* shared secret keys (*S*_1_, *S*_2_, …, *S_m_*). PKI VM*_master_* is responsible for assigning each shared secret key to the *m* mapper VM*_i_* computers.

When the system is destroyed, the newly appointed PKI VM*_master_* only needs to collect *n* shared secret keys to restore the original system key of *S*. After passing through the threshold to share the secret operation, each mapper VM*_i_* in the cloud has the shared key, *S_i_*, and its corresponding public key, *P_i_*.

In addition, we use {M_i_, TD_sigi_} to represent the individual signature of each mapper, VM_i_, and when the PKI VM_*master*_ receives each TD_sigi_ and verifies the signatures of m VM_i_, the signature, M_i_ and TD_sigi_ (i = 1, 2, 3, …, m), are combined to form a group signature {M, TD_Sig_}, which must satisfy TD_Sig_ = TD_Sig1_ + TD_Sig2_ + TD_Sig3_ +...+ TD_Sigm_ mod p, where p is a prime. By using this step, we can confirm that the data are calculated by the correct computer VM_i_ and that the restructured data are correct.

Map operation

(1) Initially, when the PKI VM*_master_* receives the request from the user, it then decides which mapper VM*_i_* can participate in the operation in the future and then transmits the task to VM*_i_*. With the purpose of confirming the validity of the PKI VM*_master_* identity of the request, the PKI VM*_master_* is required to sign the request information sent to the mapper VM*_i_*, the identity of the PKI VM*_master_* as *ID_VMmaster_* and the data to be transmitted as *TD*_Sig*i*_, so the mapper VM*_i_* subsequently sends a request to verify the identity of the PKI VM*_master_*. Here, we represent the complete data *TD* = [H(H(H(Sig*_C_*_1_(*T_D_*_1_))|H(Sig*_C_*_2_(*T_D_*_2_)))|H(H(Sig*_C_*_3_(*T_D_*_3_))|H(Sig*_C_*_4_(*T_D_*_4_))))||(Sig*_C_*_1_(*T_D_*_1_)|Sig*_C_*_2_(*T_D_*_2_)|Sig*_C_*_3_(*T_D_*_3_)|Sig*_C_*_4_(*T_D_*_4_))], and divide *TD* into several segments, according to mapping/reduction operations.

[*Request*| *ID*_VM*master*_, *TD_Sigi_*]_VM*master*_*sig*_

Once the mapper VM*_i_* receives the signature message, it uses the VM*_master_*’s public key to verify whether the identity of the requestor VM*_master_* is correct and then signs the response message to reply, the mapper VM*_i_*’s own identity *ID*_VM*i*_*_-__mapper_*, and the transmitted message {*M*_i_, *TD*_Sig*i*_}.

[*Reply*|*ID*_VM*i*_*_-__mapper_*, {*M_i_*, *TD_Sigi_*}]_VM*i_sig*_

(2) Next, PKI VM*_master_* adopts the data segment of *TD*_Sig*i*_ as the input and computes its HMAC (*TD*_Sig*i*_) value, accompanied by the mapper VM*_i_*’s identity *ID*_VM*i*_*_-__mapper_*, the original data *TD*_Sig*i*_, the timestamp and the results of the partial group signatures {*M*_i_, *TD*_Sig*i*_}. Finally, PKI VM*_master_* signs the entirety of the data **through** the secret sharing key of *S*_i_ on the receiving end and transmits the signature result to the mapper VM*_i_*.

[*S_i_*, [*ID*_VM*i*_*_-__mapper_*, *TD_sigi_*, *Time Stamp*, {*M_i_*, *TD_Sigi_*}||HMAC(*TD_sigi_*)]*_Si_sig_*

The transmitted data received by the mapper VM*_i_* are then decrypted with the public key of P*_i_* corresponding to the secret sharing key of *S_i_*, and the correctness of the shared key of *S_i_* and the integrity of the HMAC are verified.

Reduce operation

(3) Once the reducer, VM*_x_*, accepts an appointed job from the VM*_master_*, for the purpose of ensuring that the sender is accurate, the PKI VM*_master_* must sign the requested information, the VM*_master_* identity *ID*_VM*master*_ and the **delivered** data *Inf_req_*, and the reducer of VM*_x_* will then be able to verify the identity of the VM*_master_*.

[*Request*| [*ID*_VM*master*_, *Inf_req_*] ]_VM*master_sig*_

After receiving the delivered data, VM*_x_* has to confirm whether the VM*_master_* identity named *ID*_VM*master*_ is correct through the VM*_master_* public key and subsequently signs the response reply, the reducer’s identity *ID*_VM*x*-*reducer*_ and the response data *Inf_rep_*.

[*Reply*|*ID*_VM*x*-*reducer*_, *Inf_rep_*]_VM*x*_*_-__reducer_Sig_*

(4) Successively, the reducer, VM*_x_*, receives the data segments {*M_i_*, *TD*_Sig*i*_}~{*M_n_*, *TD*_Sig*m*_ }, the timestamp, and the **sequence** number signed by the mappers VM*_i_*’s secret-sharing key S*_i_* from mappers VM*_i_* (*i* = 1~*m*).

Mappers VM_(1~*m*)_→The reducer VM*_x_*

[{*M*_i_, *TD_Sigi_*}, *Time Stamp*, *SqNo*]*_Si_*__*Sig*_

(5) After receiving the delivered data from the mapper, VM_(1~*m*)_, the VM*_x_* reducer immediately requests the corresponding *P_i_* public key to the VM*_i_* mapper from the PKI VM*_master_*.

The reducer VM*_x_*→PKI VM*_master_*

[*Request*|*P_i_*]_VM*x-reducer_Sig*_

(6) Subsequently, **the** VM*_x_* reducer gains the public key corresponding to the *S_i_* from VM*_master_*, which participates in the operation mapper, VM_(1~*m*)_.

The PKI VM*_master_*→The reducer VM*_x_*

[*Reply*|*P_i_*]_VM*master_Sig*_

After the reducer, VM*_x_*, obtains the public key corresponding to *S_i_*, the encrypted data are encrypted, and the signatures {*M_i_*, *TD*_Sig*i*_} (*i* = 1, 2, 3,..., *m*) of each VM*_i_* are merged to become the group signature, {*M*, *TD*_Sig_}, where *TD*_Sig_ = *TD*_Sig1_ + *TD*_Sig2_ + *TD*_Sig3_ + … + *TD*_Sig*m*_ mod *p*. The reducer, VM*_x_*, then uses the PKI VM*_master_*’s public key to confirm the {*M*, *TD*_Sig_} group signature and then merges the data segments into an integral message. Eventually, the VM*_x_* reducer delivers this integral information to the vehicle that sent the request to complete the map/reduce operation with a confirmed identity and secure data transmission.

The proposed mechanism is fast, effective and also fault-tolerant. When the master is damaged, only *n* mapper’s shared secret keys need to be collected to reassemble the system secret key, *S*. Additionally, the mapper and the reducer protect each other through each other’s secret shared keys to secure the transmitted data from being changed during data transmission. The time stamp and the sequence number protect against repeated reply transmissions. Moreover, by verifying the identity of the mapper/reducer and assigning work through the master, malicious computers can also be prevented from impersonating mappers or reducers to perform DoS, denial of service.

## 4. The Analysis of Security Additionally, Performance

This section presents an analysis of the security and performance of the proposed scheme. While the data are being transferred, we have to esnure the integrity of the transaction block and discover the modified block. Additionally, we also need to ensure the participant of joining secure map/reduce operations to avoid impersonal attacks. Additionally, this section provides the efficiency analysis of performing a group signature to compare it with the Kerberos scheme. The detailed descriptions are as follows:

(1) Merkle tree verification

The advantage of Merkle trees is that if a block is collapsed or altered, the root value of the Merkle tree can be gained by recomputing along the path of the destroyed node to the root node of the Merkle tree. In addition, we can also determine the location of the damaged child nodes of the Merkle tree according to the following steps, as shown by the blue line in Figure 5.

Step 1. Taking Sig*_C_*_1_(T*_D_*_1_), Sig*_C_*_2_(T*_D_*_2_), Sig*_C_*_3_(T*_D_*_3_), Sig*_C_*_4_(T*_D_*_4_) as the input, calculate the latest H* hash value for the MNL2_1_ root node and confirm if the original value [H(H(H(Sig*_C_*_1_(*T_D_*_1_))|H(Sig*_C_*_2_(*T_D_*_2_)))|H(H(Sig*_C_*_3_(*T_D_*_3_))|H(Sig*_C_*_4_(*T_D_*_4_))))||(Sig*_C_*_1_(*T_D_*_1_)|Sig*_C_*_2_(*T_D_*_2_)|Sig*_C_*_3_(*T_D_*_3_)|Sig*_C_*_4_(*T_D_*_4_))] is equal to H*. When they are not equal, proceed to verify their child nodes, MNL1_1_ and MNL1_2_.
Step 2. Similar hash jobs are performed repeatedly, and if MNL1_1_ is the same and the node MNL1_2_ is different, this study will examine the child nodes, MN_3_ and MN_4_, of the node MNL1_2_.
Step 3. Similar hash jobs are repeated, and if MN_3_ is equal but MN_4_ is not, this study will examine MN_4_ and finally realize the accurate collapsed node.

In the process of this comparison manipulation, this mechanism merly consumes the time complexity of comparison O(log_2_*M*), and *M* is the number of transaction blocks. In addition, O(*M*) of creating this Merkle tree is the amount of hash operations computed.

(2) Group signature verification

Initially, the PKI VM*_master_* announces a public key, *Z*, to the participating group members to confirm the message {*M_i_*, *TD*_Sig*i*_} of the signature. Additionally, this formula for the verification is as follows:

(1) $$Z^{TD_{Sigi}{}^{\prime }}=M^{M}g^{TD_{Sig}}\mathrm{mod}p$$

If the above verification formula (1) can be derived, it means that the group signature of the message {*M_i_*, *TD*_Sig*i*_} is correct because the VM*_i_* signature value {*M_i_*, *TD*_Sig*i*_} can be satisfied.

(2) $$Z_{i}{}^{TD_{Sigi}}{}^{\prime }\left(\prod_{j=1,j\neq i}^{n}\frac{-x_{j}}{x_{i}-x_{j}}\right)=M_{i}^{M}g^{TD_{Sigi}}\mathrm{mod}p$$

Multiply the above equation (2) *n* times (i = 1, 2, 3, …, *n*) to obtain Equation (3).

(3) $$\prod_{j=1}^{n}Z_{i}{}^{TD_{Sigi}}{}^{\prime }\left(\prod_{j=1,j\neq i}^{n}\frac{-x_{j}}{x_{i}-x_{j}}\right)=\prod_{i=1,j\neq 1}^{n}M_{i}^{M}g^{TD_{Sigi}}\mathrm{mod}p$$

Additionally,

(4) $$g^{TD_{Sigi}{}^{\prime }}\sum_{i=1}^{i}f\left(x_{i}\right)\prod_{j=1,j\neq i}^{n}\frac{-x_{i}}{x_{i}-x_{j}}\mathrm{mod}p={\left(\prod_{i=1}^{n}M_{i}\right)}^{Mg\sum_{i=1}^{n}TD_{Sigi}}\mathrm{mod}p$$

Let *X_i_* = 0, and this study can derive the following equation

$$g^{TD_{Sigi}{}^{\prime }f\left(0\right)}=M^{M}g^{TD_{Sig}}\mathrm{mod}p.$$

The correct verification equation for the signature of the verification group can be derived from the above equation.

(5) $$Z^{TD_{Sigi}{}^{\prime }}=M^{M}g^{TD_{Sig}}\mathrm{mod}p$$

(3) Efficiency evaluation

**Figure 8:**
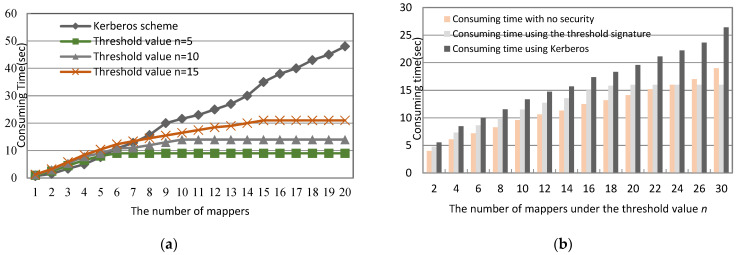
The time consumed during reconstructing the system key, *S*. (**a**) Under various threshold values. (**b**) Under various schemes and fixed threshold value.

In this study, we adopt MediaTek MT7697 CPU with ARM^®®^ Cortex^®®^-M4 with a floating-point computing unit and 1T1R 802.11 b/g/n Wi-Fi as OBUs and RSUs. In addition, this study embeds the blockchain protocol and the ECDSA cryptosystem in OBUs and RSUs to simulate the proposed multi-level blockchain management protocol. Moreover, in order to prove system efficiency and facilitate the evaluation of the time of reconstructing the system key, S, this study exploits a (*m*, *n*) threshold scheme and gradually increases the threshold value from 1 to *n* on the *m* mapper VMs to rebuild the *S* system key and evaluate the consuming time. Figure 8a indicates that in the beginning, the system increases stably and needs more time to recover the system key, *S*, with the increasing threshold values. However, when the system reaches the threshold value, the time consumed becomes smooth, as shown in Figure 8a. Additionally, when we compare our group signature scheme with no security and the Kerberos scheme, Figure 8b depicts that our proposed scheme costs more time than a no-security scheme when we increase the number of the mappers *m* under the fixed threshold value *n*. However, it is still better than the Kerberos scheme because the need for Kerberos is an authentication server and a ticket-granting server, thus consuming extra operations in cloud operations.

In addition, the limitations of the proposed method depend on the threshold value of reconstructing the system key. With the increase in the number of vehicles, Figure 8b shows that our proposed threshold signature does not change significantly compared to Kerberos’s time consumption growth. This is mainly because the system key can be reconstructed as long as we collect the partial key that reaches the threshold value, unlike Kerberos, which must obtain partial keys from all of the participants in order to reconstruct the system key. Therefore, after reaching the threshold value, our system only needs a fixed time to reconstruct the system key, and thus the communication overhead is not huge. Figure 8b shows a few changes in consuming time after collecting *n* partial keys to reconstruct the system key under a fixed threshold value of *n*.

(4) The threshold cryptosystem with fault tolerance

Since this study adopts the threshold-sharing key mechanism, it has a fault-tolerant mechanism and can avoid a single point of error. When the system key of *S* is damaged or VM*_master_* collapses. Mapper VMs collect the secret-sharing key, *S_i_*, of the surviving mappers to recover the system key, *S*, through Lagrange interpolation polynomial to figure out *S*, and then the system key can be regained and avoid the system collapsing. Even if the system faces malicious attacks, only *n* mappers VMs exit to sign the transmitted data instead of *m* members, and then the system can verify whether the transmitted data are correct.

(5) Blockchain integrity

Since the prev_hash field of the next block indicates the hash value of the previous block header, the system can use this as a certificate of overall blockchain integrity [26,27]. When an intruder modifies the historical transaction in the previous block number, *N* − 1, even if only any node value in the Merkle tree is modified, the Merkle root value of the block header will be affected by the linkage, and the prev_hash value of the subsequent block number *N* will also be invalidated, unless the intruder also changes the prev_hash value of each block header in the blockchain, but there are technical difficulties due to the decentralized nature of the blockchain.

(6) Comparison of the related research

This study compares our proposed scheme with the related research on scalability, communication cost and a single-point failure. The proposed multi-level blockchain is composed of three levels, which are responsible for blockchain operation. Therefore, when the number of vehicles is increased, the problem of excessive single-point calculation and single-point failure can be avoided. At the same time, it can reduce much of the communication costs between vehicles and make the system more scalable. By contrast, other approaches mostly use the traditional blockchain architecture. They have problems related to expansion difficulty, huge communication costs and the single-point failure of authentication, as described in Table 2.

## 5. Conclusions

Since the IoVs operates in a communication environment open to all, personal information is shared within the wireless network. Consequently, the issue of information security in terms of the IoVs will therefore be important [29,30]. The main contribution of this study is to propose a new transaction block and ECDSA digital signature that are able to ensure the non-repudiation of the transaction and assure the integrity of the transaction data. In addition, the designed multi-level blockchain architecture distributes the operations within intra_clusterBC and inter_clusterBC to improve the efficiency of the entire block. Moreover, in order to secure the security of OBU-RSU-BS-VM, this research adopts ECDH key exchange agreement to protect the transmitted information. Eventually, we consider the collected data from vehicles that will be delivered to the back-end cloud service platform to perform the big data computing and analysis and generate value-added information [31,32]. This research has to ensure the VMs identity of joining the map/reduce operations, and therefore we exploit the secret-sharing mechanism to propose the group signature and threshold key protection mechanism to accomplish the secure map and reduce operations. The threshold scheme can recover the system key as long as the threshold partial key is collected. This avoids the occurrence of PKI single-point failure. Overall, the proposed architecture is capable of securing data transmission among IoV devices and cloud service platforms. In this way, this study can ensure the security of the information and obtain a secure IoV.

## Figures and Tables

**Table 1:** The usage of notation in this system.

Notations	Description
*C_i_*	The vehicle *C* is numbered *i*.
*T_Dx_*	Transaction block data *T_Dx_* transmitted by the vehicle *C_x_*.
Sig*_Cx_*(*T_Dx_*)	The vehicles *C_x_* performs an ECDSA digital signature on the transmitted transaction block data *T_Dx_*.
*R_x_*	The router *R* is numbered *x*.
H*(Sig*_Cx_*(*T_Dx_*))	Perform a SHA256 hash operation on the ECDSA digital signature of the delivered transaction block Sig*_Cx_*(*T_Dx_*), * representing the value obtained after a new hash operation.
VM*_i_*	The virtual machine VM is numbered *i*.
TS	Timestamp.
ID*_VMx_*	The identity ID of the virtual machine VM*_x_*.
EK*_K_*	The data are encrypted using the key *K*.
||	Data concatenation operations.
MNL*i_x_*	The vehicle node number *x* at the *i*th level of the Merkle tree.
*BS_x_*	The base station is numbered *x*.
MN*_x_*	The leaf node of the Merkle tree is numbered *x*
RSU*_xy_*	The road site equipment is located at level *x*, and the number is *y*.
Block*_xy_*	The block is located at level *x*, and the number is *y*.
VM*_master_*	The master VM in map/reduce operation of the cloud.
intra_clusterBC*_x_*	The intra cluster blockchain is numbered *x*.
*SK*_*R*x_*R*y_	The session key between devices *R_x_* and *R_y_*.
TD*_Sigi_*	The signature of TD through VM*_i_*.
[ ]_VM*master_sig*_	The signature of [ ] through VM*_master_*.
*S*	The system key *S*.
*P*	The corresponding public key *P*.
*S_i_*	The shared secret *Si*.
*P_i_*	The corresponding public key *Pi*.

**Table 2:** The comparison of the related research.

Authors	Methods	Scalability	Communication Cost	Single-Point Failure
Our scheme	A Multi-level blockchain	V	Low	No
Jiang et al. [7]	Five categories of blockchain	X	High	Yes
Ma et al. [9]	A privacy-secure and decentralized VNets	V	Low	Yes
Dai et al. [10,11]	A private blockchain	X	High	No
Lu et al. [13]	A privacy protection authentication	X	Medium	Yes
Cui et al. [14]	A consortium blockchain	X	Medium	No
Bagga et al. [15]	A batch authentication protocol	V	Medium	No
Maria et al. [16]	An anonymous authentication	X	Medium	Yes
Zheng et al. [17]	A lightweight authentication	V	Medium	No
Song et al. [28]	An anonymous authentication	V	Medium	No

## Data Availability

Not applicable.
